# Risk Stratification of Thyroid Nodules Using Ultrasound Cine-Loop Video Sequences

**DOI:** 10.3390/cancers17162616

**Published:** 2025-08-09

**Authors:** Tabea Nikola Schmidt, Martin Freesmeyer, Christian Kühnel, Falk Gühne, Larissa Rosenbaum, Robert Drescher, Philipp Seifert

**Affiliations:** Clinic of Nuclear Medicine, Jena University Hospital, 07749 Jena, Germany

**Keywords:** thyroid nodules, ultrasound, Cine loop, TIRADS, thyroid cancer

## Abstract

Background/Objectives: Static image captures (SICs) are the prevailing methodology for documenting thyroid nodules (TNs) on ultrasound examinations. Ultrasound cine-loop (CL) video sequences of the thyroid enable the storage and review of the entire organ in PACS, analogous to sectional imaging modalities such as CT or MRI. Expanding on SIC, the collection of more extensive datasets is possible, with the potential to enhance diagnostic performance. However, there is an absence of reliable data concerning this process. Methods: This retrospective, tertiary care, single-center study included all patients with cytologically and histopathologically diagnosed TNs from 01/16 to 12/23. A standardized CL protocol was routinely acquired in addition to conventional SIC. The diagnostic performance of ACR and Kwak TIRADS was examined for both CL and SIC in a PACS. Advantages, challenges, and limitations of CL were analyzed. Conclusions: In total, 189 patients with 329 TNs (78% females, aged 54 ± 15 years; 76% diagnosed via surgery; 14% malignant) were included. On SIC, 58 TNs (18%) were not identified (all benign). When comparing CL with SIC, a strong correlation was observed for nearly all ultrasound features (echogenicity, composition, margin, and foci; each *p* < 0.001) and both TIRADSs (each *p* < 0.001). The diagnostic accuracy of CL was slightly superior, with maximum values of 85% for ACR and 87% for Kwak TIRADS, respectively. Rating confidence and image quality exhibited superiority on SIC (each *p* < 0.001). The occurrence of image artifacts was more prevalent in CL (*p* < 0.001). The integration of cine loops into thyroid ultrasound was found to be a seamless process, thereby enhancing the risk stratification of nodules. Image quality impairments manifested more frequently in cine loops, while static image captures demonstrated higher levels of assessment confidence.

## Simple Summary

Standardized ultrasound cine-loop video sequences can be seamlessly integrated into the clinical workflow, enabling the precise risk stratification of thyroid nodules via review in a PACS. The integration of additional cine loops into the clinical routine has been demonstrated to exhibit high levels of acceptance, whilst also enabling a comprehensive evaluation of the entire thyroid gland, including all potentially malignant thyroid nodules. The diagnostic accuracy of thyroid nodule risk stratification can be enhanced using different Thyroid Imaging Reporting and Data Systems when comparing the results of cine-loop assessments with those of static image captures. Nevertheless, cine-loop evaluation is more complex than evaluating static images; larger datasets are generated and need to be contextualized. Furthermore, the existence of a greater number of artifacts has been demonstrated to result in a decline in the confidence of subjective assessments.

## 1. Introduction

Thyroid nodules (TNs) show a high prevalence, especially in iodine-deficient regions [1,2]. While most are benign, accurate risk stratification is essential to avoid unnecessary fine-needle cytology (FNC) or diagnostic surgery approaches while ensuring dependable detection rates of thyroid carcinomas [1,3]. For this purpose, ultrasound remains the primary imaging tool [4,5]. Since laboratory findings can be inconspicuous even in cases of histopathologically diagnosed thyroid carcinoma, imaging and associated risk stratification protocols are highly relevant [6]. These risk stratification systems (RSSs), including the Thyroid Imaging Reporting and Data System (TIRADS), have been developed to standardize the evaluation of TNs and facilitate reliable treatment courses [7,8,9,10]. However, clinical documentation typically relies on static image captures (SICs), which limit retrospective review and potentially omit crucial findings.

Conversely, the incorporation of ultrasound cine-loop (CL) video sequences facilitates the acquisition of more comprehensive image datasets that encompass the thyroid gland in its entirety, analogous to CT or MRI. These videos can be transferred to a picture archiving and communication system (PACS) for the purposes of secondary reading, teleradiology, and educational training sessions [11,12]. CLs have the potential to enhance the clinical performance of ultrasound investigations and, consequently, the risk stratification of TNs. In other medical disciplines, such as cardiology and prenatal care, the utilization of CL is prevalent [13,14,15,16].

Our tertiary care nuclear medicine clinic specializes in diagnostics of thyroid disease and is a founding member of the German TIRADS study group (GTSG, www.tirads.de, accessed on 13 July 2025). In February 2015, we incorporated additional CLs into all routine ultrasound examinations of the neck. The methodology demonstrated significant clinical relevance in the post-treatment follow-up of differentiated thyroid carcinomas [17]. The initial CL pilot studies evaluating TNs yielded encouraging results in the preoperative context. These studies demonstrated that CL can be reliably evaluated when ultrasound videos are recorded by non-physician personnel [12]. Moreover, training and experience with TIRADS appear to influence interobserver variability in the risk stratification of TNs based on CL [18].

The present study aimed to build on these findings by evaluating the diagnostic accuracy and applicability of RSS for TNs based on CL in a large cohort of cytologically and histopathologically diagnosed TNs. The primary objectives were to obtain clinical acceptance of additional CL images to determine the reliability of video images in identifying and risk-stratifying clinically relevant thyroid nodules and to identify the challenges posed by extensive datasets in assessing thyroid lesions in a real-world scenario.

## 2. Materials and Methods

### 2.1. Study Design and Patients

This retrospective, tertiary care, single-center study included all patients with cytologically and/or histopathologically diagnosed TNs who had previously undergone CL examination between January 2016 and December 2023.

The following inclusion criteria are defined as follows:

- The CL of TNs available on the PACS (transverse and sagittal planes);
- Ultrasound performed <6 months prior to FNC/surgery;
- Cytological and/or histopathological results of TNs available (Bethesda II without subsequent surgery and Bethesda III, IV, and V with subsequent surgery).

The exclusion criteria were as follows:

- A history of radioiodine therapy or thyroid surgery;
- Ultrasound > 6 months before FNC/surgery;
- No CLs available (external pre-interventional ultrasound);
- Inadequate CL techniques (e.g., missing transverse or sagittal scans);
- Inadequate ultrasound image quality (images were not reasonably assessable);
- Inconclusive final diagnosis (e.g., missing histopathological results after ambiguous FNC);
- Bethesda I as the final result;
- Bethesda III/IV/V without subsequent surgery;
- Thyroid carcinoma detected in metastases, and no primary tumor identified in the thyroid gland;
- The localization of cytologically/histopathologically diagnosed TN was not relatable to TNs on ultrasound.

**Figure 1:**
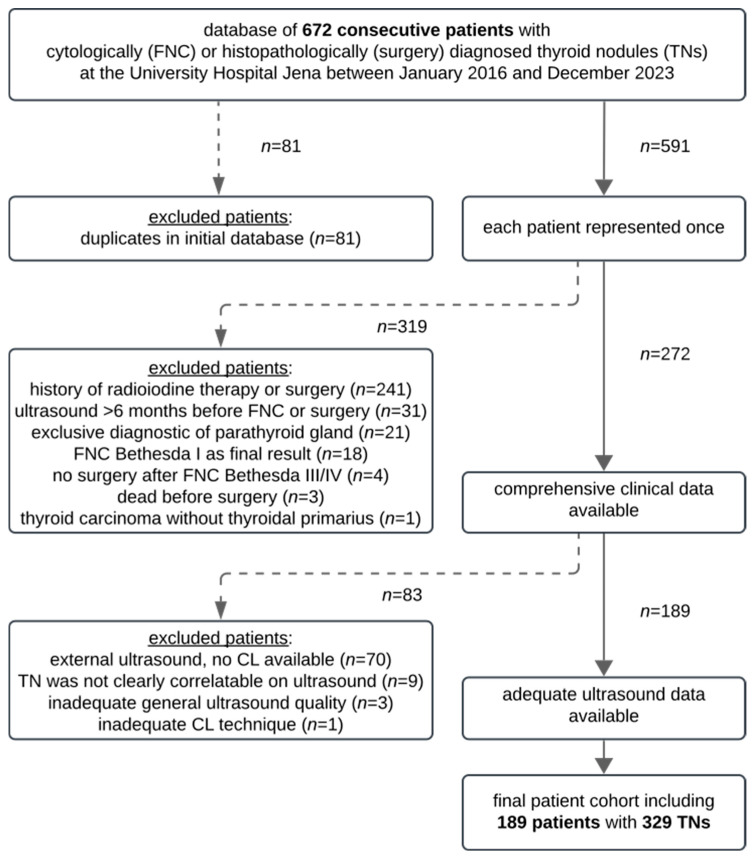
Flowchart of patient selection.

More details of patient selection are demonstrated in the flowchart in Figure 1.

### 2.2. Ultrasound Examinations

In February 2015, all routine thyroid ultrasound examinations, conducted by trained physicians only, included CLs according to a local SOP in addition to the standard SIC documentation [11,12]. Briefly, four separate CL scans were performed in the following predefined order:

- 1: Right-sided medial thyroid compartment; transverse plane; and cranio-caudal movement.
- 2: Median thyroid compartment, transverse plane and cranio-caudal movement.
- 3: Left-sided medial thyroid compartment; transverse plane; and cranio-caudal movement.
- 4a/4b: Thyroid compartment; sagittal plane; right–left; and possibly two single scans (depending on the prominence of the larynx).

**Figure 2:**
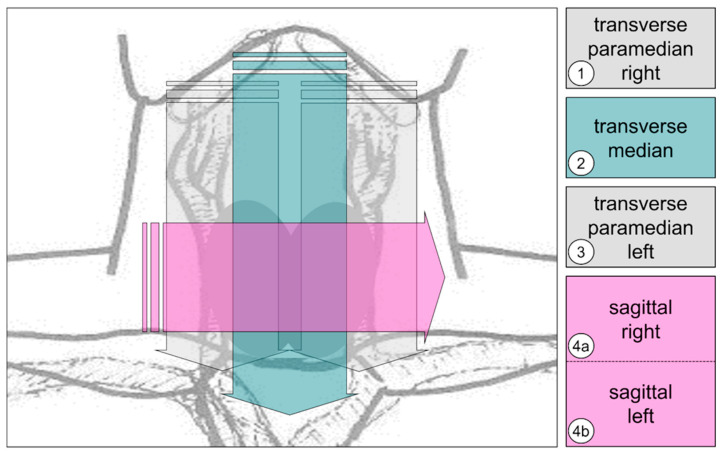
SOP of CL acquisition.

Subsequent transfer was performed for all CL and SIC data to the PACS. A short schematic overview is presented in Figure 2. A video showing CL acquisition is provided in Supplemental 1. The LOGIQ E9 ultrasound device (GE Medical Systems, Milwaukee, WI, USA) was used. The purpose of SOP was to ensure gapless coverage of the entire thyroid gland. Technical ultrasound parameters, such as the number and localization of foci or the ultrasound frequency, were individually optimized for each patient to adequately assess the thyroid gland.

### 2.3. Data Assessment

Clinical data comprised sex, age, medical history, thyroid status (organ size, number of TNs), thyroid medication, previous treatments, iodine intake, and laboratory tests. Cytological/histopathological reports were comprehensively reviewed to ensure correct lesion identification on ultrasound in the case of multiple TNs. FNC results were recorded referencing the 2017 Bethesda system [19]. For multinodular thyroid glands without surgery, only cytologically diagnosed TNs with Bethesda II were included.

The review of both SIC and CL was conducted by a single observer, who underwent one year of rigorous training from a TIRADS expert, revising more than 5000 ultrasound images. Complex cases were discussed with an acknowledged TIRADS expert with over 12 years of experience from a study center and a publication history of over 50 thyroid-related articles over the past decade. All evaluations were carried out on a PACS viewer. Initially, CLs were reviewed, followed by SIC with a 3-month interval to prevent recognition bias. The cytological/histopathological results were withheld during ultrasound assessment. Factors considered for TN evaluation were localization, diameter, volume, ultrasound features, and risk stratification according to the American College of Radiology (ACR) and Kwak TIRADS [7,9]. The overall level of assessment confidence was documented using a 4-point ordinal scale (ranging from very confident and confident to ambiguous and uncertain).

Technical aspects, such as the number and localization of ultrasound foci, artifacts, general image quality, and integrity, were recorded. Additional parameters assessed for CLs were the number of image frames per loop and the number of frames capturing respective TNs.

### 2.4. Data Analyses and Statistics

Data were recorded on Excel software (Version 2016, Microsoft Corporation, Redmond, WA, USA, last access: 24 May 2025) and transferred to IBM SPSS Statistics software (Version 28.0, Armonk, NY, USA, https://www.ibm.com/de-de/products/spss, last access: 28 May 2025) as well as RStudio (Version 2024.12.0, Posit-Software, PBC, Boston, MA, USA, https://posit.co/download/rstudio-desktop/, last access: 31 May 2025) for statistical analyses.

Kolmogorov–Smirnov tests (distribution analyses), Chi-square tests, Spearman’s correlation coefficient, positive predictive value (PPV), negative predictive value (NPV), sensitivity (SENS), specificity (SPEC), and diagnostic accuracy (ACC) were used. *p* < 0.05 was considered significant.

Spearman’s correlation coefficients were labeled “weak” for values ranging from 0.200 to 0.299, “moderate” for values ranging from 0.300 to 0.399, “strong” for values ranging from 0.400 to 0.699, and “very strong” for correlations > 0.700 [20].

The best cutoffs (benign versus malignant) were calculated for ACR and Kwak TIRADS. For ACR TIRADS, TR4 was subdivided into TR4 (4 pts.), TR4 (5 pts.), and TR4 (6 pts.). For Kwak TIRADS, 4C was subdivided into 4C (3 pts.) and 4C (4 pts.).

## 3. Results

### 3.1. Patient Data and Clinical Characteristics

Clinical integration of CL into the workflow of thyroid ultrasound was a seamless process. Acquisition of the additional video scans was completed within an average time frame of approximately 1 minute. A total of 329 TNs in 189 patients were included (42 males, 22.1%; 147 females, 77.8%; aged 15–82 years, mean age: 54 ± 15 years). Volumes of the thyroids ranged from 3 to 196 mL (mean: 35 ± 30 mL), including 115 multinodular (60.8%), 46 uninodular (24.3%), and 28 binodular (14.8%) thyroids. TNs were located in the right lobe in 164 (49.8%), in the left lobe in 121 (36.8%), and in the isthmus in 44 cases (13.4%). The diameters of the included TNs ranged from 5 to 97mm (mean: 26 ± 14 mm). Of these TNs, 32 had a diameter of ≤10 mm (9.7%), 282 were benign (85.7%) and 47 were malignant (14.3%).

For 96 TNs (29.2%), cytological results were available, conducted by two pathological institutes, presenting as follows: 1 Bethesda I (1.0%), 78 Bethesda II (81.3%), 12 Bethesda III/IV (12.5%), 2 Bethesda V (2.1%), and 3 Bethesda VI (3.1%). Of these, 17 TNs (5.2%) were subsequently histopathologically diagnosed during surgery (1 Bethesda I, 12 Bethesda III/IV, 2 Bethesda V, 2 Bethesda VI). In one case of Bethesda VI, colorectal carcinoma metastasis was diagnosed but not resected. For 250 TNs (76.0%), histopathological results from seven hospitals and their respective pathological institutes were available.

The malignant TNs were classified as follows: thirty-three papillary thyroid carcinoma (70.2%), containing three papillary microcarcinomas, seven follicular thyroid carcinoma (14.9%), four metastases of non-thyroid primary tumors (8.5%), two anaplastic thyroid carcinoma (4.3%), and one insular variant of a poorly differentiated thyroid carcinoma (2.1%) [21].

Of the included TNs, 58 (17.6%) were not documented on SIC. All of these were proven to be benign, and 89.7% of cases occurred during resections of multinodular thyroids. The average diameter of these TNs was 24 mm; nine of which were ≤10 mm (15.5%). Consequently, only 271 TNs were subjected to further evaluation on SIC, while all 329 TNs were assessed on CL.

### 3.2. Ultrasound Features

A strong correlation was identified between CL and SIC for most ultrasound features, including composition, echogenicity, margin, and echogenic foci (r > 0.400; *p* < 0.001). A weak correlation was observed for shape (r = 0.380; *p* < 0.001). On CL, the presence of very hypoechoic TNs (*p* = 0.013) and smooth margins (*p* < 0.001) was seen less often, while ill-defined margins were observed with higher frequency (*p* < 0.001). Comprehensive data are shown in Table 1.

### 3.3. Risk Stratification Systems

For both RSSs investigated, strong correlations were found between CL and SIC (each r > 0.400, *p* < 0.001). The statistical analyses revealed no significant differences between the results. Overall assessment confidence showed a weak correlation between CL and SIC (r = 0.239, *p* < 0.001) and was superior for SIC with significant differences in the categories "very confident" and "ambiguous" (each *p* < 0.001). Further details are displayed in Table 2.

The optimal cutoff values for distinguishing between benign and malignant TNs were determined at ACR TR5 and Kwak 4C, with an accuracy of >80% for both CL and SIC. Despite a marginally superior accuracy for Kwak 4C (4 pts.), the cutoff was determined at Kwak 4C (3 pts.) on account of the very low sensitivity of <10% for Kwak 4C (4 pts.). All the calculated values are shown in Table 3.

### 3.4. Technical Ultrasound Features

Table 4 presents detailed results of the artifacts assessed for the respective TNs on CL versus SIC. On CL, artifacts were more common (*p* < 0.001). Apart from acoustic shadowing, the same ultrasound planes were affected.

CL contains multiple individual images (frames) arranged into a video sequence. The number of frames ranged from 46 to 393 (mean: 132 ± 51) in the transverse plane, and from 33 to 360 (112 ± 44) in the sagittal plane. The number of frames capturing designated TN ranged from 10 to 188 (42 ± 28) in the transverse plane, and from 8 to 281 (53 ± 32) in the sagittal plane. The number of frames per CL is primarily determined by the pace of the transducer movement. Slower movement generated more frames and thereby higher image quality, but also a larger data size (~3.5 gigabytes per CL, ~0.3 gigabytes per SIC). Fewer CL frame rates frequently caused blurring (responsible for 33 of 90 “bad image quality” assessments).

On CL, the number of foci applied was 1–4 (2.9 ± 0.5). On SIC, in transverse (sagittal) planes, 3.6% (4.6%) of the TNs had no focus covering them. A total of 45.9% (44.7%) of the TNs were captured by at least one focus in the transverse (sagittal) plane. Furthermore, 29.5% (29.2%) of the TNs were fully captured by every existing focus in the transverse (sagittal) plane. On CL, 4.9% (4.3%) of the TNs were not captured by any focus in the transverse (sagittal) plane. In 74.5% (73.9%) of the cases, at least one focus was located within the TN in the transverse (sagittal) plane, while in 16.4% (17.3%) of instances, every placed focus encompasses the TN. No significant differences were observed regarding the coverage of foci for the investigated TNs between CL and SIC.

## 4. Discussion

Archiving SIC remains the prevailing method for thyroid ultrasound in clinical practice; however, it has limitations, particularly the investigator’s selective documentation [4,18,22]. CL addresses this by enabling a comprehensive review on PACS, reducing information loss [23]. In this study, all patients received additional thyroid CL, with only one case missing a sagittal scan. Consequently, over an eight-year period with multiple investigators, this approach was accepted due to its efficiency and seamless clinical integration, resulting in only approximately one minute of additional time expenditure [12,17].

CL combines the benefits of sectional imaging with ultrasound technology, enhancing diagnostic accuracy, particularly for TN risk stratification. The study found that 17.6% of the investigated TNs were detected solely on CL. Nevertheless, these manifestations were deemed to be of negligible clinical significance (all benign) and manifested most commonly in multinodular glands. Consequently, it can be assumed that the ultrasound investigators may have pre-filtered the TNs as unworthy of documentation.

To standardize thyroid ultrasound, several RSSs for TNs emerged, ensuring consistent terminology, especially for less experienced investigators [8,24,25,26,27,28]. However, no RSS has achieved a leading position [10,29,30]. This study assessed the ACR TIRADS (the most comprehensive system) and the Kwak system (chosen for its simplicity) using CL in comparison to SIC [7,9].

Strong correlations were revealed between CL and SIC for ACR and Kwak TIRADS. Differences included less frequent documentation of very hypoechoic echogenicity on CL, likely due to the identification of individual more echogenic frames, and a higher rate of ill-defined margins, probably due to blurred images (which will be discussed below).

Diagnostic accuracies for both RSSs were high (~85%) on CL, surpassing SIC. However, this study highlights a general disadvantage of RSSs: many borderline TNs fall into ACR TR4 and Kwak 4C categories [28]. According to ACR recommendations, all TR4 TNs ≥ 15 mm should be referred to FNC, and those ≥10 mm should be referred to follow-up [7]. TR4 can be subdivided into TR4 (4 pts.), TR4 (5 pts.), and TR4 (6 pts.), Kwak 4C into 4C (3 pts.), and 4C (4 pts.), with each additional point in these classifications potentially increasing the malignancy potential [7,9,31,32]. Higher ACC values for TR4 (5 pts.) and TR4 (6 pts.) suggest improved cutoff-based classifications. However, in concordance with other studies, we found the best ACC values for conventional cutoffs at ACR TR5 and Kwak 4C [29,33].

Assessment confidence was higher for SIC, likely due to a lower number of images and the sequential order of assessments (CL first). A learning effect may have increased confidence in evaluating SIC. Additionally, artifacts can affect ultrasound diagnostics [34]. They occur more frequently on CL (80% of artifact types), which may cause higher intra- and interobserver variability [18,35]. The acquisition of assessable ultrasound images relies on meticulous parameter adjustments (e.g., frequency, foci, penetration depth, and amplification), which are easier to obtain for single-image SIC than for 100–180 frames per CL [11]. The number of frames per loop, determined by the movement speed of the ultrasound transducer, relevantly affects CL [12].

**Figure 3:**
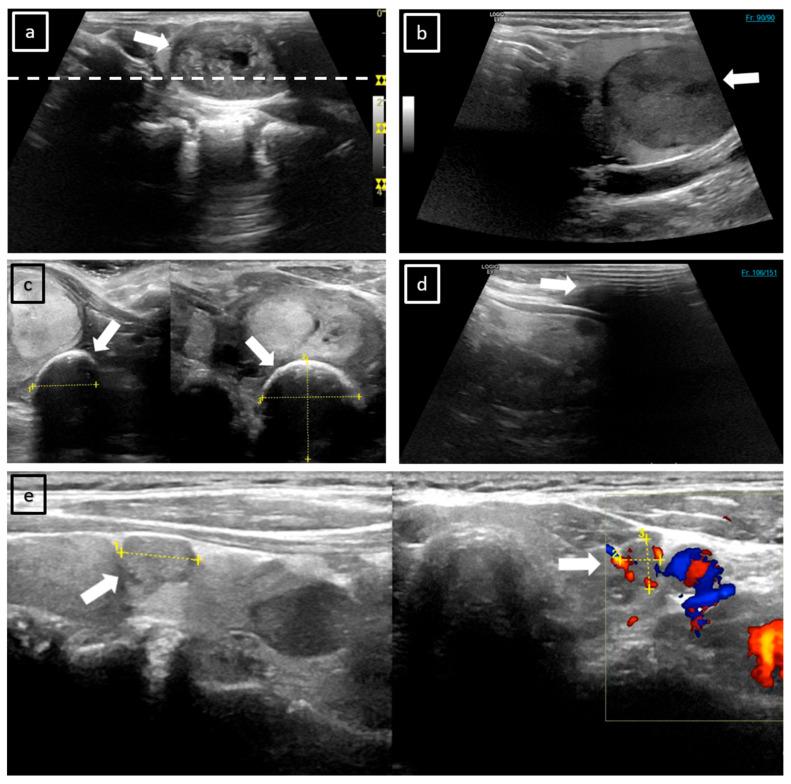
Examples of frequent ultrasound image artifacts. (**a**) The SIC of a left-sided TN (white arrow) in the sagittal plane. On the right side of the image, the depths of three foci are visible as yellow markers. The white dotted line indicates that only one of these foci lies within the TN, which may result in a reduction in image quality in the region of interest. (**b**) This illustration depicts a prevalent issue that manifests particularly in caudally localized TNs on CL. The TN is cut in the sagittal plane, as indicated by the white arrow. It is important to note that this TN has not been fully captured by the respective sagittal CL scan, as frame 90 of 90 is reached (numbers are displayed in the upper right corner). (**c**) TNs in the transverse and sagittal plane on SIC, with superimposed measurement lines (yellow markers). The assessment is hindered by extensive rim calcification at the top of the TN (marked by white arrows), which obscures its structural ultrasound features and impairs the evaluation of its size. (**d**) Acoustic shadowing (white arrow) of the primary components of the displayed CL capture is shown, likely attributable to an inadequate application of ultrasound gel. (**e**) The left part of this SIC shows the sagittal plane with measurement lines (yellow markers) superimposed upon the TN (white arrow). The right part of the image depicts additional Doppler visualization, which complicates the assessment (white arrow).

Cropped poles, breathing-related movements, and inadequate application of ultrasound gel (Figure 3) can also impair CL quality. In contrast, CL can effectively avoid acoustic shadowing by allowing the selection of unobscured frames. In the future, the quality of CL could be enhanced by implementing a more comprehensive training program for all physicians performing ultrasound procedures with automated artifact detection and correction algorithms. Physician training programs may be based on previously acquired scans, thereby facilitating the identification of prevalent issues and ensuring a targeted, effective resolution. To ensure the integrity of the data, personnel could be requested to undertake a direct examination of the recorded CL to identify artifacts and be instructed to re-initiate the acquisition process if necessary. In cases where patients present special anatomical features, such as a short neck or a prominent larynx, it is imperative to ensure the application of a sufficient amount of ultrasound gel. Furthermore, the proper positioning of the head and neck region is crucial for acquiring high-quality ultrasound data.

From this perspective, the documentation of the CL can be a valuable training component, as it enables the instruction of investigators using their own images, thereby facilitating a more hands-on and personalized learning experience. Additionally, CL conserves personnel resources as video sequences do not require acquisition by a physician [12].

The use of the LOGIQ E9 ultrasound device requires manual focus adjustments that commonly misalign with the TNs, particularly in multinodular thyroids [12]. Newer generations, such as LOGIQ E10, feature autofocus technology and consistent image quality across the entire field of view. Technological advancements will significantly support the implementation of CL into future clinical routines.

In everyday clinical practice, B-mode ultrasound, with other diagnostic tools, such as elastography and Tc-99m scintigraphy, comprehensively assesses TNs. FNA still remains the gold standard for the preoperative diagnosis of TNs [36]. However, this invasive method and potentially unnecessary surgical interventions can be avoided through accurate risk stratification involving the aforementioned tools and FDG- or I-124-PET/CT scans, which are being increasingly performed in higher-level clinical settings [37,38].

Scintigraphy is a valuable diagnostic tool in the identification of hyperfunctioning TNs and, therefore, ruling out malignancy in suspicious cases [39,40]. To employ scintigraphy in a profitable manner, it is helpful to carry out image correlation with ultrasound images to ensure that a correct spatial assignment of the suspicious TN is made for the FNA [37]. The superimposition of ultrasound data and metabolic imaging, resulting in Tc-99m-pertechnetate-SPECT/ultrasound and I-124-PET/ultrasound fusion imaging, facilitates more precise evaluations of diminutive or unfavorably located TNs [37]. Given CL’s capacity to enhance diagnostic precision and generate a more comprehensive dataset on thyroid gland morphology, it can be hypothesized that, in conjunction with metabolic imaging, CL facilitates the more precise localization of TNs, necessitating further evaluation compared to SIC use alone.

Despite artifacts and technical limitations, CL slightly outperformed SIC’s diagnostic accuracy, highlighting the potential of this novel methodology. Addressing technical constraints, such as the relatively large amounts of data (10–15 times of SIC), could enhance its clinical utility.

AI integration may further refine CL by identifying additional ultrasound features and morphological patterns. Prior research has demonstrated SIC’s efficacy in AI applications [41]. In 2019, Wildman-Tobriner et al. demonstrated that an AI-optimized version of TIRADS exhibited higher diagnostic accuracy and significantly higher specificity in comparison to conventional ACR TIRADS [42]. In 2021, Liu et al. showed that AI can classify fewer thyroid nodules as TR4, thereby increasing specificity and consequently reducing the number of unnecessary FNAs [43]. Similar findings were recently presented by Carnabatu et al. [44]. The ultrasound AI adapter improved PPV, only marginally impacting the NPV. The integration of the AI adapter into risk stratification protocols had the capacity to enable more precise patient counseling and to obviate the necessity for biopsies in cases where a definitive diagnosis would otherwise be difficult to reach.

In conclusion, these studies demonstrate that AI has already made a significant contribution to the diagnostic process of TNs in the setting of SIC. This finding suggests that AI can serve as an effective adjunctive tool not only for SIC but also for CL. It has the potential to enhance the already high diagnostic accuracy of TNs in video sequences.

A considerable number of deep learning-based applications are commercially available. Several studies have demonstrated their capacity to outperform physicians in assessing malignancy in TNs [42,45,46]. One of these programs is the S-Detect software (Samsung Ultrasound RS80A, Samsung Medison Co., Ltd., Seoul, South Korea, https://previous.samsunghealthcare.com/de/products/UltrasoundSystem/V8/GeneralImaging/benefit, last access: 14 July 2025). This software has been the focus of numerous studies. It utilizes TIRADS criteria to provide the diagnosis “possibly benign” or “possibly malignant” [47]. A 2022 meta-analysis, including 17 studies on S-Detect, showed that the program had high diagnostic accuracy [48].

Another application that has only recently been approved by the American Food and Drug Administration is KOIOS DS (KOIOS DS Thyroid, Version Number: 3.5, KOIOS Medical, Inc., New York, NY, USA, https://koiosmedical.com/products/koios-ds-thyroid/, last access: 14 July 2025). This AI tool employs two selected images of the designated TN, subsequently generating a description of TN characteristics based on ACR TIRADS criteria. This is followed by a recommendation either for or against FNA [49]. Evidence suggests that KOIOS AI has the potential to assist in the field of thyroid ultrasound diagnosis, with studies indicating improvements in diagnostic accuracy and a reduction in interobserver variability [45,50].

PIUR (PIUR tUS Infinity, Version Number: 3.3, PIUR Imaging DE, Munich, Germany, https://www.piurimaging.com/piur-tus-infinity, last access: 14 July 25) incorporates innovative techniques, including multiplanar reconstruction and voxel-based volume measurements based on tomographic ultrasound [51,52]. This objective approach identifies potential malignant findings in TNs. Furthermore, the program facilitates the execution of ACR TIRADS calculations, which may lead to re-evaluation in instances of divergent opinions. Three-dimensional visualization can help contextualize TNs within the thyroid parenchyma, enabling a more comprehensive evaluation of the structural characteristics and other features deemed pertinent for TIRADS assessment [53].

Determining whether these AI applications can also be used with CL or contingent on the usage of SIC is a subject for further investigation. Programs such as PIUR already enable a more comprehensive evaluation through broader image data, outlining the benefits of using CL in thyroid diagnostics.

### Limitations

The relatively high malignancy rate of 14.3% indicates selection bias, which might result from the inclusion criteria pertaining to cytologically/histopathologically diagnosed TNs, in conjunction with the iodine deficiency of the study center’s respective catchment areas. This potentially diminishes the generalizability of our findings and underscores the necessity to consider the patient cohort and the region where the CL should be utilized in the future. It may be advisable to investigate the prevalence of thyroid cancer in patient cohorts undergoing investigations with this method. In view of the information obtained, further research with patient cohorts exhibiting lower prevalences of thyroid cancer is a logical next step. This research could determine whether the performance of this new method is, at least in part, contingent on the higher malignancy rate shown by this study’s cohort.

CL acquisitions may have varied by examiner, potentially affecting the angles in the different planes and settings of the ultrasound device, and no interobserver data can be reported. Thus, future studies are required to evaluate the interdependence of the results on the observer’s expertise.

In addition, the selection of the trained observer could have influenced the results, as a more experienced observer might improve the outcomes, irrespective of the ultrasound method. The study by Schenke et al. (2024) provides evidence to support this hypothesis. In their research, twelve observers evaluated twenty TNs; the results indicated that the outcome was heavily influenced by the observer’s level of experience [18].

The data used to evaluate the two methods was deemed suitable for analysis, as the selection process involved excluding ultrasound datasets exhibiting substandard image quality. This may have introduced bias, as in clinical routines, substandard quality images cannot always be ruled out when evaluation is required for diagnostic purposes.

Another potential bias may pertain to the exclusion criterion of patient history with radioiodine therapy or thyroid surgery, which can cause substantial changes in thyroid parenchyma and mitigate the evaluation of TNs in CL. Therefore, further research is required on this new ultrasound method with pretreated patients for thyroid diagnostics.

## 5. Conclusions

Ultrasound CL of the thyroid gland facilitates comprehensive sectional image data storage of the entire organ in PACS. This methodology enables the precise identification of clinically relevant lesions and yields superior diagnostic accuracy in the risk stratification of thyroid nodules compared to conventional static image captures. Despite the diminished subjective confidence in TIRADS classifications, which is primarily attributed to more frequent image artifacts and the significantly increased amount of data generated, the clinical advantages outweigh this drawback. The integration of CL into the clinical ultrasound workflow can be executed seamlessly according to the introduced SOP. It is recommended that clinics with experience in thyroid diagnostics employ this approach.

## Figures and Tables

**Table 1:** Ultrasound features on CL and SIC.

Ultrasound Features	CL (*n* = 329)	SIC (*n* = 271)	Chi^2^	Spearman’s Correlation
Composition				
Cystic or almost completely cystic	59 (17.9)	39 (14.4)	*p* = 0.243	r = 0.657 *p* < 0.001
Spongiform	1 (0.3)	1 (0.4)	*p* = 0.891	
Mixed cystic and solid	123 (37.4)	120 (44.3)	*p* = 0.087	
Solid or almost completely solid	146 (44.4)	111 (41.0)	*p* = 0.400	
Echogenicity				
Anechoic	24 (7.3)	10 (3.7)	*p* = 0.057	r = 0.574 *p* < 0.001
Hyperechoic or isoechoic	129 (39.2)	97 (35.8)	*p* = 0.390	
Hypoechoic	157 (47.7)	133 (49.1)	*p* = 0.741	
Very hypoechoic	19 (5.8)	31 (11.4)	*p* = 0.013	
Shape				
Wider than tall	300 (91.2)	234 (86.3)	*p* = 0.059	r = 0.380 *p* < 0.001
Taller than wide	29 (8.8)	37 (13.7)	*p* = 0.059	
Margin				
Smooth	103 (31.3)	136 (50.2)	*p* < 0.001	r = 0.406 *p* < 0.001
Ill-defined	205 (62.3)	112 (41.3)	*p* < 0.001	
Lobulated or irregular	21 (6.4)	23 (8.5)	*p* = 0.325	
Extra-thyroidal extension	0 (0.0)	0 (0.0)	- *	
Echogenic Foci				
None or large comet-tail artifacts	245 (74.5)	203 (74.9)	*p* = 0.902	r = 0.523 *p* < 0.001
Macrocalcifications	51 (15.5)	43 (15.9)	*p* = 0.902	
Peripheral (rim) calcifications	6 (1.8)	10 (3.7)	*p* = 0.158	
Punctate echogenic foci	27 (8.2)	15 (5.5)	*p* = 0.202	

* No *p*-value could be calculated with zero results for this category in CL and SIC.

**Table 2:** ACR and Kwak TIRADS classification and assessment of confidence for CL and SIC.

Result	CL (*n* = 329)	SIC (*n* = 271)	Chi^2^	Spearman’s Correlation
ACR TIRADS				
TR1	23 (7.0)	10 (3.0)	*p* = 0.078	r = 0.475 *p* < 0.001
TR2	37 (11.2)	37 (11.2)	*p* = 0.372	
TR3	89 (27.1)	60 (18.2)	*p* = 0.166	
TR4	142 (43.2)	125 (38.0)	*p* = 0.467	
TR4 (4 pts.)	81 (24.6)	84 (31.0)	*p* = 0.082	
TR4 (5 pts.)	36 (10.9)	29 (10.7)	*p* = 0.925	
TR4 (6 pts.)	25 (7.6)	12 (4.4)	*p* = 0.108	
TR5	38 (11.6)	39 (11.8)	*p* = 0.301	
Kwak TIRADS				
3	65 (19.8)	51 (15.5)	*p* = 0.772	r = 0.569 *p* < 0.001
4A	107 (32.5)	84 (25.5)	*p* = 0.690	
4B	124 (37.7)	103 (31.3)	*p* = 0.936	
4C	33 (10.0)	33 (10.0)	*p* = 0.403	
4C (3 pts.)	28 (8.5)	27 (8.2)	*p* = 0.540	
4C (4 pts.)	5 (1.5)	6 (1.8)	*p* = 0.528	
5	0 (0.0)	0 (0.0)	- *	
Assessment Confidence				
Very confident	120 (36.5)	143 (43.5)	*p* < 0.001	r = 0.239 *p* < 0.001
Confident	163 (49.5)	116 (35.3)	*p* = 0.100	
Ambiguous	41 (12.5)	11 (3.3)	*p* < 0.001	
Uncertain	5 (1.5)	1 (0.3)	*p* = 0.156	

* No *p*-value could be calculated with zero results for this category in CL and SIC.

**Table 3:** Different cutoffs (benign vs. malignant) for ACR and Kwak TIRADS (CL *n* = 329, SIC *n* = 271).

Cutoff	Ultrasound Method	SENS	SPEC	PPV	NPV	ACC
ACR TIRADS						
TR4 TR4 (4 pts.)	CL	68.1	47.5	17.8	89.9	50.5
	SIC	76.7	42.9	22.0	89.7	48.7
TR4 (5 pts.)	CL	51.1	73.4	24.2	90.0	70.2
	SIC	57.4	76.3	33.8	89.5	73.1
TR4 (6 pts.)	CL	40.4	58.8	30.2	89.5	78.1
	SIC	44.7	86.6	41.2	88.2	79.3
TR5	CL	36.2	92.6	44.7	89.7	84.5
	SIC	34.0	89.7	41.0	86.6	80.1
Kwak TIRADS						
4C 4C (3 pts.)	CL	37.2	94.3	48.5	86.6	85.4
	SIC	34.9	92.8	45.5	89.5	81.5
4C (4 pts.)	CL	9.3	100.0	80.0	86.7	86.6
	SIC	9.3	100.0	66.7	83.8	83.4

**Table 4:** Ultrasound image artifacts.

	Ultrasound Method		Affected Ultrasound Plane		
	CL (*n* = 658) *	SIC (*n* = 542) *	More Frequently in (CL)	More Frequently in (SIC)	Chi^2^
No artifacts	406 (61.7)	433 (79.9)	transverse	transverse	*p* < 0.001
Bad image quality	90 (13.7)	32 (5.9)	transverse	transverse	*p* < 0.001
Indistinguishable TNs	105 (16.0)	51 (9.4)	sagittal	sagittal	*p* < 0.001
TNs cut/interfered	50 (7.6)	14 (2.6)	sagittal	sagittal	*p* < 0.001
Acoustic shadowing	7 (1.1)	12 (2.2)	sagittal	equally	*p* = 0.112

* Values of *n* = 658 and *n* = 542 derived from two examined US planes.

## Data Availability

The data are contained within the article or Supplementary Material.

## Supplementary Materials

The following supporting information can be downloaded at: https://www.mdpi.com/article/10.3390/cancers17162616/s1, Video S1: Video of CL acquisition according to local SOP with directions of ultrasound probe movement and orientation on the patient’s neck.

## Abbreviations

The following abbreviations are used in this manuscript:

ACC	diagnostic accuracy
ACR	American College of Radiology
AI	artificial intelligence
CL	cine loops (video sequences on ultrasound)
FNC	fine-needle cytology
NPV	negative predictive value
PACS	Picture Archiving and Communication System
PPV	positive predictive value
pts.	points
RSSs	risk stratification systems
SENS	sensitivity
SICs	static image captures
SPEC	specificity
SOP	standard operating procedure
TIRADS	Thyroid Imaging Reporting and Data System
TN(s)	thyroid nodule(s)

## References

[1] Kobaly K., Kim C.S., Mandel S.J. (2022). Contemporary Management of Thyroid Nodules. Annu. Rev. Med..

[2] Russ G., Leboulleux S., Leenhardt L., Hegedus L. (2014). Thyroid incidentalomas: Epidemiology, risk stratification with ultrasound and workup. Eur. Thyroid. J..

[3] Alexander E.K., Cibas E.S. (2022). Diagnosis of thyroid nodules. Lancet Diabetes Endocrinol..

[4] Boers T., Braak S.J., Rikken N.E.T., Versluis M., Manohar S. (2023). Ultrasound imaging in thyroid nodule diagnosis, therapy, and follow-up: Current status and future trends. J. Clin. Ultrasound.

[5] Raposo L., Freitas C., Martins R., Saraiva C., Manita I., Oliveira M.J., Marques A.P., Marques B., Rocha G., Martins T. (2022). Malignancy risk of thyroid nodules: Quality assessment of the thyroid ultrasound report. BMC Med. Imaging.

[6] Gambardella C., Offi C., Clarizia G., Romano R.M., Cozzolino I., Montella M., Di Crescenzo R.M., Mascolo M., Cangiano A., Di Martino S. (2019). Medullary thyroid carcinoma with double negative calcitonin and CEA: A case report and update of literature review. BMC Endocr. Disord..

[7] Tessler F.N., Middleton W.D., Grant E.G., Hoang J.K., Berland L.L., Teefey S.A., Cronan J.J., Beland M.D., Desser T.S., Frates M.C. (2017). ACR Thyroid Imaging, Reporting and Data System (TI-RADS): White Paper of the ACR TI-RADS Committee. J. Am. Coll. Radiol..

[8] Russ G., Bonnema S.J., Erdogan M.F., Durante C., Ngu R., Leenhardt L. (2017). European Thyroid Association Guidelines for Ultrasound Malignancy Risk Stratification of Thyroid Nodules in Adults: The EU-TIRADS. Eur. Thyroid. J..

[9] Kwak J.Y., Han K.H., Yoon J.H., Moon H.J., Son E.J., Park S.H., Jung H.K., Choi J.S., Kim B.M., Kim E.K. (2011). Thyroid imaging reporting and data system for US features of nodules: A step in establishing better stratification of cancer risk. Radiology.

[10] Chen Q., Lin M., Wu S. (2022). Validating and Comparing C-TIRADS, K-TIRADS and ACR-TIRADS in Stratifying the Malignancy Risk of Thyroid Nodules. Front. Endocrinol..

[11] Seifert P., Kuhnel C., Reissmann I., Winkens T., Freesmeyer M. (2024). Standardized acquisition and documentation of cine loops on conventional thyroid ultrasound. Laryngorhinootologie.

[12] Seifert P., Maikowski I., Winkens T., Kuhnel C., Guhne F., Drescher R., Freesmeyer M. (2021). Ultrasound Cine Loop Standard Operating Procedure for Benign Thyroid Diseases-Evaluation of Non-Physician Application. Diagnostics.

[13] Thomas K., Burke L., McGettigan M. (2023). Use of cine images in standard ultrasound imaging: A survey of sonologists. Abdom. Radiol..

[14] Wei Y., Yang B., Wei L., Xue J., Zhu Y., Li J., Qin M., Zhang S., Dai Q., Yang M. (2024). Real-time carotid plaque recognition from dynamic ultrasound videos based on artificial neural network. Ultraschall Med..

[15] Scott T.E., Jones J., Rosenberg H., Thomson A., Ghandehari H., Rosta N., Jozkow K., Stromer M., Swan H. (2013). Increasing the detection rate of congenital heart disease during routine obstetric screening using cine loop sweeps. J. Ultrasound Med..

[16] Zhen C., Wang H., Cheng J., Yang X., Chen C., Hu X., Zhang Y., Cao Y., Ni D., Huang W. (2023). Locating Multiple Standard Planes in First-Trimester Ultrasound Videos via the Detection and Scoring of Key Anatomical Structures. Ultrasound Med. Biol..

[17] Sopuschek M.P., Freesmeyer M., Winkens T., Kuhnel C., Petersen M., Guhne F., Werner A., Seifert P. (2024). Standard operating procedure (SOP) for cervical ultrasound cine loop video sequences in the follow-up of differentiated thyroid carcinoma (DTC). Endocrine.

[18] Schenke S.A., Petersen M., Gorges R., Ruhlmann V., Zimny M., Richter J.P., Groener D., Baumgarten J., Kreissl M.C., Stahl A.R. (2024). Interobserver Agreement in Ultrasound Risk Stratification Systems for Thyroid Nodules on Static Images Versus Cine-Loop Video Sequences. Diagnostics.

[19] Cibas E.S., Ali S.Z. (2017). The 2017 Bethesda System for Reporting Thyroid Cytopathology. Thyroid.

[20] Akoglu H. (2018). User’s guide to correlation coefficients. Turk. J. Emerg. Med..

[21] Fat I., Kulaga M., Dodis R., Carling T., Theoharis C., Rennert N.J. (2011). Insular variant of poorly differentiated thyroid carcinoma. Endocr. Pract..

[22] Suzuki S., Midorikawa S., Fukushima T., Shimura H., Ohira T., Ohtsuru A., Abe M., Shibata Y., Yamashita S., Suzuki S. (2015). Systematic determination of thyroid volume by ultrasound examination from infancy to adolescence in Japan: The Fukushima Health Management Survey. Endocr. J..

[23] Dormagen J.B., Gaarder M., Drolsum A. (2015). Standardized cine-loop documentation in abdominal ultrasound facilitates offline image interpretation. Acta Radiol..

[24] Ha E.J., Moon W.J., Na D.G., Lee Y.H., Choi N., Kim S.J., Kim J.K. (2016). A Multicenter Prospective Validation Study for the Korean Thyroid Imaging Reporting and Data System in Patients with Thyroid Nodules. Korean J. Radiol..

[25] Chung S.R., Ahn H.S., Choi Y.J., Lee J.Y., Yoo R.E., Lee Y.J., Kim J.Y., Sung J.Y., Kim J.H., Baek J.H. (2021). Diagnostic Performance of the Modified Korean Thyroid Imaging Reporting and Data System for Thyroid Malignancy: A Multicenter Validation Study. Korean J. Radiol..

[26] Seifert P., Gorges R., Zimny M., Kreissl M.C., Schenke S. (2020). Interobserver agreement and efficacy of consensus reading in Kwak-, EU-, and ACR-thyroid imaging recording and data systems and ATA guidelines for the ultrasound risk stratification of thyroid nodules. Endocrine.

[27] Seifert P., Schenke S., Zimny M., Stahl A., Grunert M., Klemenz B., Freesmeyer M., Kreissl M.C., Herrmann K., Gorges R. (2021). Diagnostic Performance of Kwak, EU, ACR, and Korean TIRADS as Well as ATA Guidelines for the Ultrasound Risk Stratification of Non-Autonomously Functioning Thyroid Nodules in a Region with Long History of Iodine Deficiency: A German Multicenter Trial. Cancers.

[28] Piticchio T., Russ G., Radzina M., Frasca F., Durante C., Trimboli P. (2024). Head-to-head comparison of American, European, and Asian TIRADSs in thyroid nodule assessment: Systematic review and meta-analysis. Eur. Thyroid. J..

[29] Kim D.H., Chung S.R., Choi S.H., Kim K.W. (2020). Accuracy of thyroid imaging reporting and data system category 4 or 5 for diagnosing malignancy: A systematic review and meta-analysis. Eur. Radiol..

[30] Gao L., Xi X., Jiang Y., Yang X., Wang Y., Zhu S., Lai X., Zhang X., Zhao R., Zhang B. (2019). Comparison among TIRADS (ACR TI-RADS and KWAK- TI-RADS) and 2015 ATA Guidelines in the diagnostic efficiency of thyroid nodules. Endocrine.

[31] Huh S., Yoon J.H., Lee H.S., Moon H.J., Park V.Y., Kwak J.Y. (2021). Comparison of diagnostic performance of the ACR and Kwak TIRADS applying the ACR TIRADS’ size thresholds for FNA. Eur. Radiol..

[32] Studen K.B., Domagala B., Gaberscek S., Zaletel K., Hubalewska-Dydejczyk A. (2024). Diagnosing and management of thyroid nodules and goiter-current perspectives. Endocrine.

[33] Yang L., Li C., Chen Z., He S., Wang Z., Liu J. (2023). Diagnostic efficiency among Eu-/C-/ACR-TIRADS and S-Detect for thyroid nodules: A systematic review and network meta-analysis. Front. Endocrinol..

[34] Choi S.H., Kim E.K., Kim S.J., Kwak J.Y. (2014). Thyroid ultrasonography: Pitfalls and techniques. Korean J. Radiol..

[35] de Carlos J., Garcia J., Basterra F.J., Pineda J.J., Dolores Ollero M., Toni M., Munarriz P., Anda E. (2024). Interobserver variability in thyroid ultrasound. Endocrine.

[36] Mileva M., Stoilovska B., Jovanovska A., Ugrinska A., Petrushevska G., Kostadinova-Kunovska S., Miladinova D., Majstorov V. (2018). Thyroid cancer detection rate and associated risk factors in patients with thyroid nodules classified as Bethesda category III. Radiol. Oncol..

[37] Freesmeyer M., Winkens T., Weissenrieder L., Kuhnel C., Guhne F., Schenke S., Drescher R., Seifert P. (2020). Fusion iENA Scholar Study: Sensor-Navigated I-124-PET/US Fusion Imaging versus Conventional Diagnostics for Retrospective Functional Assessment of Thyroid Nodules by Medical Students. Sensors.

[38] Winkens T., Seifert P., Hollenbach C., Kuhnel C., Guhne F., Freesmeyer M. (2019). The FUSION iENA Study: Comparison of I-124-PET/US Fusion Imaging with Conventional Diagnostics for the Functional Assessment of Thyroid Nodules by Multiple Observers. Nuklearmedizin.

[39] Schenke S., Seifert P., Zimny M., Winkens T., Binse I., Gorges R. (2019). Risk Stratification of Thyroid Nodules Using the Thyroid Imaging Reporting and Data System (TIRADS): The Omission of Thyroid Scintigraphy Increases the Rate of Falsely Suspected Lesions. J. Nucl. Med..

[40] Richter D., Beck M., Muller S.K., Iro H., Koch M., Sievert M. (2024). Thyroid nodules as an incidental finding : Value of sonography and scintigraphy in primary diagnostics. HNO.

[41] Potipimpanon P., Charakorn N., Hirunwiwatkul P. (2022). A comparison of artificial intelligence versus radiologists in the diagnosis of thyroid nodules using ultrasonography: A systematic review and meta-analysis. Eur. Arch. Otorhinolaryngol..

[42] Wildman-Tobriner B., Buda M., Hoang J.K., Middleton W.D., Thayer D., Short R.G., Tessler F.N., Mazurowski M.A. (2019). Using Artificial Intelligence to Revise ACR TI-RADS Risk Stratification of Thyroid Nodules: Diagnostic Accuracy and Utility. Radiology.

[43] Liu Y., Feng Y., Qian L., Wang Z., Hu X. (2023). Deep learning diagnostic performance and visual insights in differentiating benign and malignant thyroid nodules on ultrasound images. Exp. Biol. Med..

[44] Carnabatu C.J., Fetzer D.T., Tessnow A., Holt S., Sant V.R. (2025). Avoidable biopsies? Validating artificial intelligence-based decision support software in indeterminate thyroid nodules. Surgery.

[45] Fernandez Velasco P., Perez Lopez P., Torres Torres B., Delgado E., de Luis D., Diaz Soto G. (2024). Clinical Evaluation of an Artificial Intelligence-Based Decision Support System for the Diagnosis and American College of Radiology Thyroid Imaging Reporting and Data System Classification of Thyroid Nodules. Thyroid.

[46] Bodoque-Cubas J., Fernandez-Saez J., Martinez-Hervas S., Perez-Lacasta M.J., Carles-Lavila M., Pallares-Gasulla R.M., Salazar-Gonzalez J.J., Gil-Boix J.V., Miret-Llaurado M., Aulinas-Maso A. (2025). Integrating Artificial Intelligence in Thyroid Nodule Management: Clinical Outcomes and Cost-Effectiveness Analysis. J. Clin. Endocrinol. Metab..

[47] Zhou L., Zheng L.L., Zhang C.J., Wei H.F., Xu L.L., Zhang M.R., Li Q., He G.F., Ghamor-Amegavi E.P., Li S.Y. (2023). Comparison of S-Detect and thyroid imaging reporting and data system classifications in the diagnosis of cytologically indeterminate thyroid nodules. Front. Endocrinol..

[48] Zhong L., Wang C. (2022). Diagnostic accuracy of S-Detect in distinguishing benign and malignant thyroid nodules: A meta-analysis. PLoS ONE.

[49] Wildman-Tobriner B., Taghi-Zadeh E., Mazurowski M.A. (2022). Artificial Intelligence (AI) Tools for Thyroid Nodules on Ultrasound, from the AJR Special Series on AI Applications. AJR Am. J. Roentgenol..

[50] Barinov L., Jairaj A., Middleton W.D., Beland Kirsch J., Filice R.W., Reverter J.L., Arguelles I., Grant E.G. (2023). Improving the Efficacy of ACR TI-RADS Through Deep Learning-Based Descriptor Augmentation. J. Digit. Imaging.

[51] Kronke M., Eilers C., Dimova D., Kohler M., Buschner G., Schweiger L., Konstantinidou L., Makowski M., Nagarajah J., Navab N. (2022). Tracked 3D ultrasound and deep neural network-based thyroid segmentation reduce interobserver variability in thyroid volumetry. PLoS ONE.

[52] Munsterman R. (2023). Deep Learning Segmentation of 3D Ultrasound Thyroid Scans. Master’s Thesis.

[53] Cheng A., Lee J.W.K., Ngiam K.Y. (2023). Use of 3D ultrasound to characterise temporal changes in thyroid nodules: An in vitro study. J. Ultrasound.

